# Comparison of serum Farnesoid X Receptor (FXR) levels and their associations with obesity-related metabolic parameters: a clinical study

**DOI:** 10.1186/s12902-026-02231-z

**Published:** 2026-04-15

**Authors:** Zeyneb Irem Yuksel Salduz, Rana Turgut, Zeynep Seval Coskun, Ozge Pasin, Murat Kartal, Aclan Ozder

**Affiliations:** 1 Department of Family Medicine, Faculty of Medicine, Bezmialem Vakıf University, Istanbul, Türkiye; 2 Department of Nutrition and Dietetics, Faculty of Health Sciences, Istanbul Kent University, Istanbul, Türkiye; 3 Department of Nutrition and Dietetics, Bezmialem Vakıf University Hospital, Istanbul, Türkiye; 4 Department of Biostatistics and Medical Informatics, Faculty of Medicine, University of Health Sciences, Istanbul, Türkiye; 5 Department of Pharmacognosy, Faculty of Pharmacy, Bezmialem Vakıf University, Istanbul, Türkiye; 6 Department of Family Medicine, Cerrahpaşa Faculty of Medicine, Istanbul University-Cerrahpaşa, Istanbul, Türkiye

**Keywords:** Bile acids, Farnesoid X Receptor (FXR), Metabolic disorders, Nutrition, Obesity

## Abstract

**Background:**

Farnesoid X Receptor (FXR) regulates the enterohepatic circulation of bile acids and influences nutrient metabolism. These functions position FXR as a potential therapeutic target for obesity and related conditions, although its association with BMI in healthy individuals remains inadequately defined.

**Objective:**

To investigate serum FXR levels in normal-weight, overweight, and obese individuals, considering their sociodemographic profiles, anthropometry, physical activity frequency, sleep patterns, eating behaviors, and laboratory findings.

**Methods:**

This prospective cross-sectional study included 80 healthy participants (43 women [53.75%] and 37 men [46.25%]) who presented to the Family Medicine Clinic or Nutrition and Diet Polyclinic at Bezmialem Vakıf University Faculty of Medicine Hospital between January 2024 and June 2024. Participants were categorized into four groups including 20 individuals based on body mass index (BMI); Group 1: 18.5–24.9 kg/m² (normal weight), Group 2: 25–29.9 kg/m² (overweight), Group 3: 30–34.9 kg/m² (obesity class I), Group 4: 35–39.9 kg/m² (obesity class II). Participants completed the Three-Factor Eating Questionnaire (TFEQ) to assess dietary habits. Biochemical parameters and FXR levels were analyzed from blood samples. All statistical analyses were conducted using SPSS version 26.0, with statistical significance defined as *p* < 0.05.

**Results:**

Gender distribution and lifestyle factors did not differ significantly between groups. Higher BMI was associated with increased levels of glucose (*p* = 0.018), HOMA-IR (*p* = 0.008), LDL (*p* = 0.011), triglycerides (*p* = 0.013), and WBC (*p* = 0.015). Emotional eating scores in Group 1 were significantly lower than in Group 4 (*p* = 0.046). The mean serum FXR level was 10.93 ± 9.56 ng/mL, with significant differences across BMI groups (*p* = 0.014), being higher in obese individuals. FXR levels were positively associated with a family history of obesity (*p* = 0.031), the use of topical agents (*p* = 0.024), and serum zinc levels (*p* = 0.025), whereas no significant associations were observed with gender (*p* = 0.721) or eating behavior scores (cognitive restraint, *p* = 0.483; uncontrolled eating, *p* = 0.581; emotional eating, *p* = 0.814).

**Conclusions:**

The observed associations between serum FXR levels and obesity-related metabolic parameters suggest that FXR may be involved in the metabolic dysregulation accompanying increased adiposity. Although experimental and translational studies have implicated FXR in glucose and lipid homeostasis, the present findings provide only preliminary clinical evidence and do not establish causality. Accordingly, FXR should be regarded as a mechanistic pathway of interest rather than a confirmed therapeutic or preventive target. Further longitudinal and interventional studies are needed to determine whether modulation of FXR activity or monitoring serum FXR levels has practical clinical relevance in obesity management.

**Supplementary Information:**

The online version contains supplementary material available at 10.1186/s12902-026-02231-z.

## Introduction

Obesity is currently recognized as the fifth leading cause of mortality worldwide. If current trends continue, an estimated 57.8% of the global population will be classified as overweight or obese by 2030. Obesity is a major risk factor for several comorbidities, including Type 2 Diabetes Mellitus (T2DM), cardiovascular disease, and dyslipidemia [1, 2]. The most commonly used criterion for defining obesity is the body mass index (BMI). According to the World Health Organization (WHO), individuals with a BMI below 18.5 kg/m² are classified as underweight, those with a BMI of 18.5–24.9 kg/m² as normal weight, 25.0–29.9 kg/m² as overweight, and ≥ 30.0 kg/m² as obese [1]. Although obesity is attributable to genetic mutations in a very small subset of individuals, it is largely a preventable condition. Current treatment strategies include medical nutrition therapy, behavioral interventions, increased physical activity, pharmacological treatments, and surgical procedures. However, the effectiveness of pharmacological treatments remains controversial, highlighting the necessity for more effective therapeutic strategies to prevent and manage obesity [3].

In recent years, bile acids have been identified as key regulators with hormone-like effects on glucose, lipid, and energy metabolism. They also play essential roles in fat and fat-soluble vitamin absorption and cholesterol regulation. The Farnesoid X Receptor (FXR), a member of the nuclear hormone receptor superfamily, is critical for bile acid and cholesterol homeostasis [4, 5]. Activation of FXR regulates the expression of several genes responsible for producing proteins that regulate the synthesis, transport, and metabolism of bile acids. FXR contributes not only to the regulation of bile acid homeostasis but also functions as a central pathway in the metabolism and distribution of nutrients and pharmaceuticals. While FXR is primarily expressed in the liver and intestine—key components of the enterohepatic circulation—it is also found in white adipose tissue, kidney, adrenal gland, stomach, pancreas, endothelial cells, and vascular smooth muscle cells. This widespread distribution underscores the diverse biological functions of FXR [5, 6].

Bile acid research has advanced notably in recent years. Talavera et al. found that total bile acid levels are higher in obese individuals and positively correlate with BMI, independent of T2DM and Nonalcoholic Fatty Liver Disease (NAFLD) [7]. Given the regulatory role of FXR in whole-body metabolism, it is considered a potential therapeutic target for obesity and its associated comorbidities [3]. Using an animal model of FXR deficiency, Cario et al. demonstrated a direct role of FXR in adipocyte function; yet, the exact molecular mechanisms remain unclear [8]. Likewise, experimental models of obesity in mice have demonstrated that FXR deficiency may exert beneficial effects on body weight regulation and glucose homeostasis [9, 10]. Mueller et al. also investigated bile acid treatment in morbidly obese patients with NAFLD and reported a reduction in FXR activation [11].

The role of FXR in the regulation of glucose, lipid, and energy metabolism, as well as its effects on improving glucose sensitivity and obesity, has been the focus of numerous reviews in recent years [12]. In this context, the study by Ding et al., highlighted FXR as the primary bile acid receptor controlling bile acid synthesis in the liver and demonstrated that FXR antagonists may be effective in alleviating high-fat diet–induced obesity and insulin resistance in mouse models [13]. Notably, activation of hepatic FXR signaling can contribute to the amelioration of metabolic diseases by reducing lipogenesis and suppressing gluconeogenesis [14]. Analyses of human liver biopsy samples have further shown that FXR activity is initially higher in individuals with obesity, and that treatment with the FXR agonist obeticholic acid markedly enhances fatty acid oxidation, mitochondrial function, and antioxidant responses, thereby significantly improving clinically desirable substrate utilization in obesity [15]. Collectively, evidence indicates that both hepatic FXR activation and intestinal FXR inhibition exert beneficial effects on obesity-related metabolic disorders [16]. Accordingly, dysregulation of bile acid metabolism and FXR signaling along the gut–liver axis contributes to the development of metabolic diseases, including obesity, diabetes, and non-alcoholic fatty liver disease [17]. In line with these findings, Jiang et al. found a positive correlation between FXR signaling and BMI in human distal ileum biopsies, suggesting that intestinal FXR inhibition may represent a novel therapeutic strategy for metabolic disorders [18]. Similarly, Tang et al., demonstrated that early-life exposure to emulsifiers increases susceptibility to obesity in mouse offspring via the gut microbiota–FXR axis [19]. Supporting the mechanistic importance of FXR, Ryan et al., reported that the metabolic benefits of vertical sleeve gastrectomy are closely associated with increased circulating bile acids and FXR activity, whereas the absence of FXR markedly attenuates surgery-induced weight loss and improvements in glucose tolerance [20]. In this context, the high expression of FXR in both the liver and intestine underscores the need to clarify tissue-specific roles in metabolic dysfunction, particularly obesity. Nevertheless, further studies are required to identify serum bile acids, related metabolites, and gut microbial taxa as biomarkers for the treatment and prevention of obesity [21]. Despite these findings, fundamental clinical studies are still needed to investigate the relationship between FXR and BMI in healthy, intervention-free individuals.

The aim of this study was to investigate the association between serum FXR levels and BMI in healthy and obese individuals. To the best of our knowledge, this is the first observational study to examine this association. These findings may contribute to the development of therapeutic strategies for obesity and its comorbidities through selective modulation of serum FXR levels.

## Methods

### Participants

A total of 80 male and female participants, aged 18–65 years and without chronic diseases, were recruited from the Family Medicine Clinic and Nutrition and Diet Polyclinic of Bezmialem Vakıf University Faculty of Medicine Hospital between January and June 2024. The sample size was determined by power analysis, which indicated that at least 52 participants were needed to detect the expected effect size (assuming a correlation coefficient of 0.38, a 95% confidence level, and 80% statistical power) [18]. Participants with a history of diabetes; gastrointestinal diseases (e.g., gastroesophageal reflux, celiac disease, Crohn’s disease) or gastrointestinal resection; chronic liver or gallbladder diseases; cholecystectomy; organ transplantation; neurological disorders (e.g., dementia, Alzheimer’s disease); mental retardation; cancer; chronic alcohol use; acute infections; or regular antibiotic use in the past month were excluded. Medication history was assessed through direct self-report during the clinical interview and cross-verified using electronic medical records to ensure accuracy. All participants were healthy volunteers with no history of chronic disease and no regular or recent use of systemic medications. Individuals taking medications known to influence metabolic, endocrine, inflammatory, hematological, or biochemical parameters were excluded, including antidiabetic agents, antihypertensive drugs, lipid-lowering therapies, antiplatelet or anticoagulant medications, corticosteroids, thyroid medications, hormone replacement therapy, oral contraceptives, antidepressants, antipsychotics, immunosuppressive drugs, nonsteroidal anti-inflammatory drugs, and vitamin or mineral supplements. The use of non-pharmacologically active topical agents (e.g., basic moisturizers or emollient creams with minimal systemic absorption) was permitted, as these products are not expected to exert systemic metabolic or endocrine effects. This screening approach minimized potential pharmacological confounding of laboratory measurements.

### Procedures

Participants were stratified into four groups according to WHO obesity classification using stratified random sampling, matched for age and gender: Group 1 (18.5–24.9 kg/m², normal weight), Group 2 (25.0–29.9 kg/m², overweight), Group 3 (30.0–34.9 kg/m², obesity class I), and Group 4 (35.0–39.9 kg/m², obesity class II). BMI was calculated as weight (kg) divided by height squared (m²) [1]. Sociodemographic data, anthropometric measurements, physical activity, and sleep patterns were recorded. Physical activity was evaluated through self-reported weekly exercise habits, including the type, frequency, and duration of regular physical activity. Based on these responses, participants were categorized as physically inactive (< 150 min/week of moderate activity), moderately active (150–300 min/week), or highly active (> 300 min/week), in accordance with the World Health Organization (WHO) guidelines for adult physical activity recommendations [22]. Sleep patterns were assessed using structured self-report questions regarding usual bedtime, wake-up time, total sleep duration, and subjective sleep quality over the previous month. Based on reported sleep duration, participants were classified as having adequate sleep (≥ 7 h/night) or insufficient sleep (< 7 h/night), in accordance with the recommendations of the American Academy of Sleep Medicine (AASM) [23].

Fasting venous blood samples were collected from participants after an overnight fast. Serum was separated by centrifugation (2500 g, 10 min), aliquoted, and stored at − 80 °C until analysis. Routine laboratory parameters—including FBG, LDL, TG, AST, ALT, CBC, Fe, Zn, Mg, ferritin, folate, TSH, vitamin B12, and vitamin D—were measured at the Bezmialem Vakıf University Medical Biochemistry Laboratory within one hour of collection to minimize pre-analytical variability. HOMA-IR was calculated as [fasting insulin (mIU/L) × fasting glucose (mg/dL)] / 405. CBC was analyzed using an automated hematology analyzer, biochemical parameters with a chemical auto-analyzer, and serum 25(OH)D levels with LC-MS using the Zivak 25(OH)D₂–D₃ kit.

Serum FXR levels were quantified separately at the Bezmialem Vakıf University Biochemistry Laboratory using a standardized sandwich ELISA kit (BT LAB, Shanghai, China), following the manufacturer’s instructions. Briefly, 5 mL of venous blood was collected from participants in the fasting state prior to any intervention and centrifuged at 3500 rpm for 10 min within 30 min of collection to obtain serum samples. The separated serum was aliquoted and stored at − 80 °C until analysis. All samples and reagents were equilibrated to room temperature prior to analysis. For the ELISA procedure, 50 µL of standard solution was added to the standard wells, while 40 µL of serum sample and 10 µL of anti-FXR antibody were added to the sample wells, followed by the addition of 50 µL of streptavidin–HRP to both standard and sample wells. The plate was sealed and incubated at 37 °C for 60 min, after which it was washed five times with wash buffer. According to the manufacturer, the intra-assay coefficient of variation was < 10% and the inter-assay coefficient of variation was < 12%. This standardized methodology ensured accuracy and reliability across all participants.

### The three-factor eating questionnaire

The Three-Factor Eating Questionnaire (TFEQ) was originally developed by Stunkard and Messick in 1985 with 51 items to assess three dimensions of eating behavior [24]. Although first applied in obese populations, its validity has also been confirmed in non-obese individuals [25]. To improve practicality, Karlsson et al. introduced the reduced 18-item version (TFEQ-R18), which includes three subscales [26]. The Three-Factor Eating Questionnaire (TFEQ-R18) assesses three dimensions of eating behavior: Cognitive Restraint (CR) — the deliberate restriction of food intake to control weight; Uncontrolled Eating (UE) — the tendency to overeat due to loss of control, often triggered by hunger; and Emotional Eating (EE) — eating in response to emotions such as stress, anxiety, or sadness [27]. The questionnaire comprises 18 items rated on a 4-point Likert scale (1 = definitely false, 4 = definitely true). Raw subscale scores (CR, UE, EE) are summed and converted to a 0–100 scale using the formula:

$$\begin{aligned}\boldsymbol{(raw \,score}-&\boldsymbol{lowest \,possible\, raw\, score }\\&/\boldsymbol{possible\, raw\, score\, range)}\times100\end{aligned}$$

Higher scores on each subscale indicate greater levels of the respective eating behaviors. The half-scale method was used to adjust for missing data, which averaged 0.06 items per participant (range: 0–2) in this study. The Turkish version of the TFEQ-R18 was validated by Kıraç et al. [28], and this validated version was used in the present study to assess eating behaviors. The full questionnaire is provided in the Supplementary Materials.

### Statistical analysis

Descriptive statistics were reported as frequency (n) and percentage (%) for qualitative variables, and as mean, median, standard deviation (SD), minimum, and maximum for quantitative variables. Normality of quantitative variables was assessed using the Shapiro–Wilk test, and homogeneity of variances was evaluated with the Levene test. For comparisons across more than two independent groups, one-way ANOVA was used when parametric assumptions were met, and the Brown–Forsythe test was applied when variances were unequal. Tukey’s post hoc test was conducted for pairwise comparisons following ANOVA. Group comparisons were additionally adjusted for age and sex using analysis of covariance (ANCOVA), and age- and sex-adjusted p values are reported where applicable. Associations between quantitative variables were assessed using Spearman’s correlation analysis. A p-value < 0.05 was considered statistically significant. All analyses were performed using SPSS software (Version 26.0; IBM Corp., Armonk, NY, USA).

## Results

### Population characteristics

The study group included 43 females (53.75%) and 37 males (46.25%). Baseline clinical parameters of the 80 participants are presented in Table 1.

**Table 1:** The clinical and laboratory parameters for all participants

Parameters	Mean ± Standard Deviation	Median [Min-Max]
Age (yr)		27 [18–55]
Waist Circumference (cm)		96 [65–135]
BMI (kg/m²)*	29.49 ± 5.69	
FBG (mg/dL)		90.53 [76–118]
HOMA-IR (mg/dL)		2.18 [0.60–10.10]
LDL (mg/dL)*	109.67 ± 29.36	
TG (mg/dL)		105.5 [38–455]
AST (U/L)		18 [12–80]
ALT (U/L)		20 [7–48]
WBC (×10³/µL)*	6.99 ± 1.54	
NLR		1.79 [0.67–4.79]
RBC (×10⁶/µL)*	4.84 ± 0.46	
HGB (g/dL)*	14.03 ± 1.42	
HCT (%)		41.05 [32.5–51.9]
MCV (fL)*	86.35 ± 4.21	
PLT (×10³/µL)*	262.11 ± 58.10	
MPV (fL)*	10.25 ± 0.86	
Zn (µg/dL)*	97.54 ± 15.91	
Fe (µg/dL)*	79.49 ± 31.73	
Mg (mg/dL)*	1.92 ± 0.13	
Ferritin (µg/L)		36.4 [2.14–234.26]
Folate (µg/L)*	8.92 ± 1.91	
TSH (mIU/L)		2 [0.71–6.93]
Vitamin B12 (ng/L)		288 [173–688]
Vitamin D (ng/mL)*	23.10 ± 10.92	
FXR (ng/mL)		6.22 [0.05–31.74]

*Normal distribution is assumed

Abbreviations: BMI, Body Mass Index; FBG, Fasting Blood Glucose; HOMA-IR, Homeostatic Model Assessment of Insulin Resistance; LDL, Low-Density Lipoprotein; TG, Triglyceride; AST, Aspartate Aminotransferase; ALT, Alanine Aminotransferase; WBC, White Blood Cell; NLR, Neutrophil Lymphocyte Ratio; RBC, Red Blood Cell; HGB, Haemoglobin; HCT, Haematocrit; MCV, Mean Corpuscular Volume; PLT, Platelet; MPV, Mean Platelet Volume; Zn, Serum Zinc; Fe, Serum Iron; Mg, Serum Magnesium; TSH, Thyroid-Stimulating Hormone; FXR, Farnesoid X Receptor

**Fig. 1:**
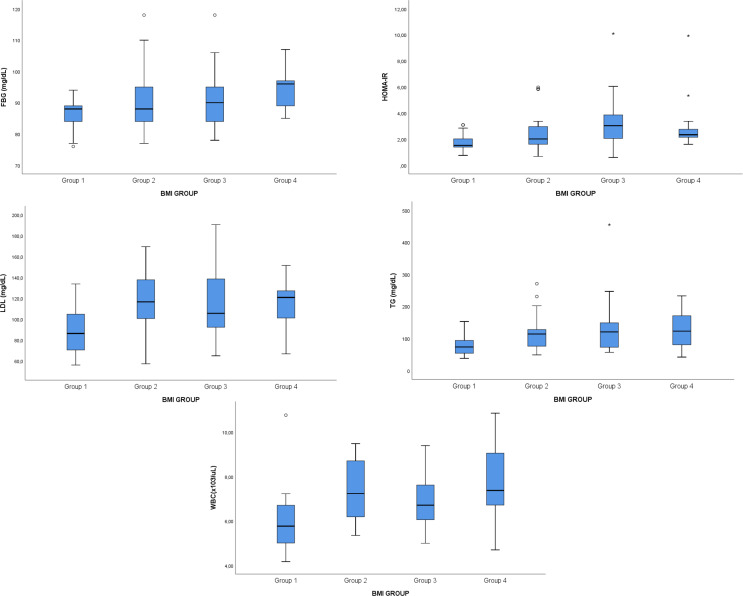
Error bar plots of FBG, HOMA-IR, lipid parameters, and WBC by BMI group. Error bar plots illustrate mean values with standard deviation for fasting blood glucose (FBG), homeostatic model assessment of insulin resistance (HOMA-IR), low-density lipoprotein cholesterol (LDL), triglycerides (TG), and white blood cell count (WBC) across body mass index (BMI) groups. BMI groups were defined as normal weight (18.5–24.9 kg/m²), overweight (25.0–29.9 kg/m²), obesity class I (30.0–34.9 kg/m²), and obesity class II (35.0–39.9 kg/m²)

Participants were categorized into four BMI-based groups (*n* = 20 per group). Gender distribution did not differ significantly between groups (*p* = 0.985). Biomarkers compared across BMI categories, together with age- and sex-adjusted p values and effect sizes, are presented in Table 2, with corresponding error-bar plots shown in Fig. 1.

**Table 2:** Laboratory parameters across BMI groups with age- and sex-adjusted analyses and effect sizes

	BMI Groups				*p*-values	Post hoc *p*	Effect size Eta Squared	Adjusted *p*
	1.Group (18.5–24.9 kg/m^2^)	2.Group (25–29.9 kg/m^2^)	3.Group (30–34.9 kg/m^2^)	4.Group (35–39.9 kg/m^2^)				
FBG (mg/dL)	88[76–97]	89.5[82–118]	87.5[77–110]	96[79–118]	0.018	1–4 = 0.012	0.023	0.358
HOMA-IR	1.51[0.68–3.10]	2.03[0.96–5.98]	2.75[0.60–10.10]	2.60[1.55–9.93]	0.008	1–3 = 0.017 1–4 = 0.014	0.146	0.018
LDL (mg/dL)	88.95[56.1–133.7]	119.45[57.3–169.4]	114.4[64.9–190.5]	119.55[65.7–151.4]	0.011	1–2 = 0.018 1–3 = 0.028	0.135	0.029
TG (mg/dL)	73.5[38–202]	107.5[49–231]	123[57–455]	121[42–233]	0.013	1–3 ≤ 0.05 1–4 ≤ 0.05	0.132	0.017
WBC(x103/uL)	5.94[4.17–10.77]	6.83[5.35–8.99]	7.03[5–9.49]	7.24[4.7–10.86]	0.015	1–4 = 0.013	0.128	0.020
AST (U/L)	18.5[12–25]	18[12–80]	19[13–31]	16.5[13–41]	0.760			0.708
ALT (U/L)	18[7–39]	17.5[9–47]	24.5[7–48]	21.5[12–47]	0.151			0.042
NLR*	1.63 ± 0.48	2.18 ± 1.09	1.88 ± 0.66	1.79 ± 0.46	0.121			0.134
RBC (×10⁶/µL)	4.73[3.98–5.43]	4.93[3.83-6]	4.85[4.18–5.85]	4.85[4.24–5.69]	0.252			0.143
HGB (g/dL)	13.6[11.9–15.8]	13.95[12.3–16.9]	13.5[9.9–16.4]	14.2[12.6–17.4]	0.320			0.098
HCT (%)	40.7[36.1–46.1]	41.35[36.2–48.9]	40.65[32.5–47.5]	42.05[37.4–51.9]	0.223			0.067
MCV (fL)	87.25[80.7–93.9]	85.4[77.7–94.5]	84.4[73.7–94.3]	87.1[81.3–93.2]	0.137			0.140
PLT (×10³/µL)	231.5[173–324]	263[172–390]	274.5[186–380]	280.5[179–380]	0.082			0.087
MPV (fL)	9.7[8.6–11.9]	10.4[9.2–12.3]	10.2[8.9–11.7]	10.1[9–12.1]	0.307			0.335
Zn (µg/dL)	100[73–113]	102[11–127]	101[90–116]	96[68–112]	0.818			0.670
Fe (µg/dL)	88[22–170]	78[28–121]	66[12–135]	75[37–120]	0.205			0.249
Mg (mg/dL)	1.91[1.69–2.15]	1.91[1.5–2.12]	1.88[1.69–2.12]	1.98[1.72–2.26]	0.311			0.421
Ferritin (µg/L)	23.07[2.14–130.13]	51.07[4.83–205.62]	34.98[3.73–234.26]	27.62[10.92–170.64]	0.341			0.243
Folate (µg/L)	8.75[7.1–12.9]	8.6[4.6–12.6]	10.2[6.6–11.9]	8.35[4.6–13.3]	0.327			0.343
TSH* (mIU/L)	2 ± 1.02	2.36 ± 1.38	2.19 ± 0.95	2.75 ± 1.49	0.457			0.231
Vitamin B12 (ng/L)	288.5[196–688]	300.5[196–521]	261[173–498]	289[179–489]	0.813			0.759
Vitamin D (ng/mL)	23.1[7.1–36.44]	22.3[5.1–44.5]	21.97[13.1–42.37]	29.4[7.27–41.48]	0.674			0.871
Age (yr)	26[20–33]	26[20–54]	27[18–47]	34.5[21–55]	0.069			
Meal frequency*	3.15 ± 0.81	2.9 ± 0.55	2.95 ± 1.05	3.35 ± 1.18	0.473			0.523
FXR	6.73[0.05–30.21]	13.02[3.29–31.74]	4.93[3.39–30]	5.53[2.27–30]	0.014	2–3 = 0.019 2–4 = 0.032	0.160	0.016

For normally distributed variables, the mean and standard deviation were reported; for non-normally distributed variables, the median (min–max) was provided. Adjusted p values were adjusted for age and sex and are presented in the final column. *Normal distribution assumption was satisfied

Abbreviations: BMI, Body Mass Index; FBG, Fasting Blood Glucose; HOMA-IR, Homeostatic Model Assessment of Insulin Resistance; LDL, Low-Density Lipoprotein; TG, Triglycerides; WBC, White Blood Cells; AST, Aspartate Aminotransferase; ALT, Alanine Aminotransferase; NLR, Neutrophil Lymphocyte Ratio; RBC, Red Blood Cell; HGB, Haemoglobin; HCT, Haematocrit; MCV, Mean Corpuscular Volume; PLT, Platelet; MPV, Mean Platelet Volume; Zn, Serum Zinc; Fe, Serum Iron; Mg, Serum Magnesium; TSH, Thyroid-Stimulating Hormone; FXR, Farnesoid X Receptor

After adjustment for age and sex, significant group differences were observed for HOMA-IR (adjusted *p* = 0.018), LDL cholesterol (adjusted *p* = 0.029), triglycerides (adjusted *p* = 0.017), and white blood cell count (adjusted *p* = 0.020). Although fasting glucose initially differed between groups (raw *p* = 0.018), this difference did not remain significant after adjustment for age and sex (adjusted *p* = 0.358). Post-hoc analyses indicated that these differences were mainly driven by lower metabolic values in the normal-weight group (Group 1) compared with higher BMI groups, particularly obesity class II (Group 4). Effect size estimates indicated small-to-moderate group effects for the variables showing significant differences (η² range: 0.128–0.146).

According to self-reports, 25% of participants indicated a family history of obesity, which differed significantly among the groups (*p* = 0.017), with the highest prevalence in Group 4 (class II obesity; 50%). Additionally, 73.8% reported no routine use of topical agents, and 77.5% reported no additional diseases beyond those specified in the exclusion criteria, with no significant differences between groups. Regarding physical activity, 67.5% described themselves as less active, 26.3% as moderately active, and 6.3% as very active, again with no significant intergroup differences. Similarly, 61.3% reported having a regular sleep pattern, which also did not differ significantly across the groups.

### Assessment of the three-factor eating questionnaire

**Fig. 2:**
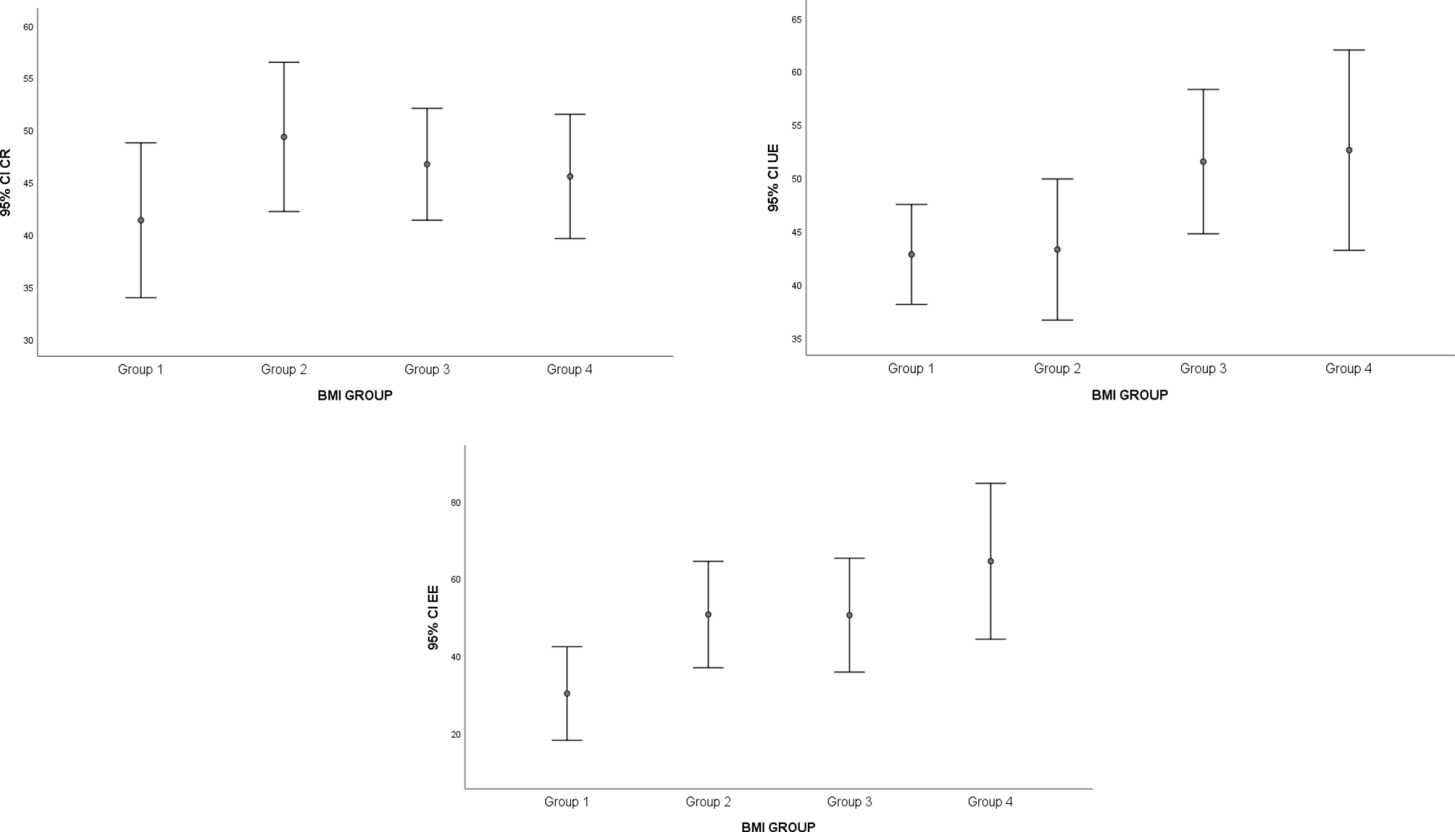
Error bar plots of eating behavior scores (CR, UE, and EE) by BMI group. Error bar plots present mean values with standard deviation for the Three-Factor Eating Questionnaire–Revised 18 (TFEQ-R18) subscales: cognitive restraint (CR), uncontrolled eating (UE), and emotional eating (EE) across body mass index (BMI) groups. BMI groups were defined as normal weight (18.5–24.9 kg/m²), overweight (25.0–29.9 kg/m²), obesity class I (30.0–34.9 kg/m²), and obesity class II (35.0–39.9 kg/m²)

The mean scores and corresponding p-values for the Three-Factor Eating Questionnaire (TFEQ-R18) responses, assessing the three dimensions of eating behavior—CR, UE, and EE—are summarized in Table 3 and error bar plots of CR, UE, EE by BMI groups were shown in Fig. 2. No statistically significant differences were observed among the groups in CR (*p* = 0.615) or UE mean scores (*p* = 0.119). However, EE mean scores differed significantly between groups (*p* = 0.042), with Group 1 showing significantly lower EE scores than Group 4 (*p* = 0.046). Additionally, a weak but significant positive correlation was found between BMI and EE scores (*r* = 0.251, *p* = 0.025), suggesting that EE scores increased with higher BMI.

**Table 3:** Mean scores of the TFEQ-R18 between groups

	1. Group	2. Group	3. Group	4. Group	*p*	Post hoc significant *p* values
BMI	(18.5–24.9 kg/m^2^)	(25–29.9 kg/m^2^)	(30–34.9 kg/m^2^)	(35–39.9 kg/m^2^)		
CR	44.4[16.67–77.78]	47.2[11.11–77.78]	44.4[27.78–77.8]	50[22.22–61.11]	0.615	
UE	44.4[25.96–55.55]	48.14[14.81–74.07]	44.4[18.51–85.18]	57.4[29.63–74.04]	0.119	
EE	22.2[0–88.8]	61.1[0–100]	50[0–100]	61.1[0–100]	0.042	
						1–4 = 0.046

*BMI, Body Mass Index; CR, Cognitive Restraint; UE, Uncontrolled Eating; EE, Emotional Eating

In the analysis of correlations between laboratory parameters and TFEQ-R18 responses, a significant but weak positive correlation was observed between blood NLR levels and CR scores (*r* = 0.294, *p* = 0.008). Similarly, PLT levels showed a significant, although weak, positive correlation with EE scores (*r* = 0.263, *p* = 0.018). Moreover, blood vitamin D levels were also positively correlated with EE scores, though weakly (*r* = 0.317, *p* = 0.043). No significant correlations were detected between other laboratory parameters and TFEQ-R18 responses.

### Assessment of the Farnesoid X Receptor

Serum FXR levels constituted the primary outcome of this study. Across all participants, the mean serum FXR concentration was 10.93 ± 9.56, and FXR levels differed significantly among the BMI-based study groups (*p* = 0.014). Specifically, post hoc analyses demonstrated that individuals in the normal-weight group exhibited significantly lower serum FXR levels compared with those in obesity class I (*p* = 0.019) and obesity class II (*p* = 0.032), indicating a stepwise increase in FXR levels with increasing adiposity.

Despite these group-based differences, Pearson correlation analysis did not reveal a significant linear association between BMI as a continuous variable and serum FXR levels (*p* = 0.095), suggesting that FXR alterations were more apparent across categorical BMI groups rather than along the continuous BMI spectrum.

**Fig. 3:**
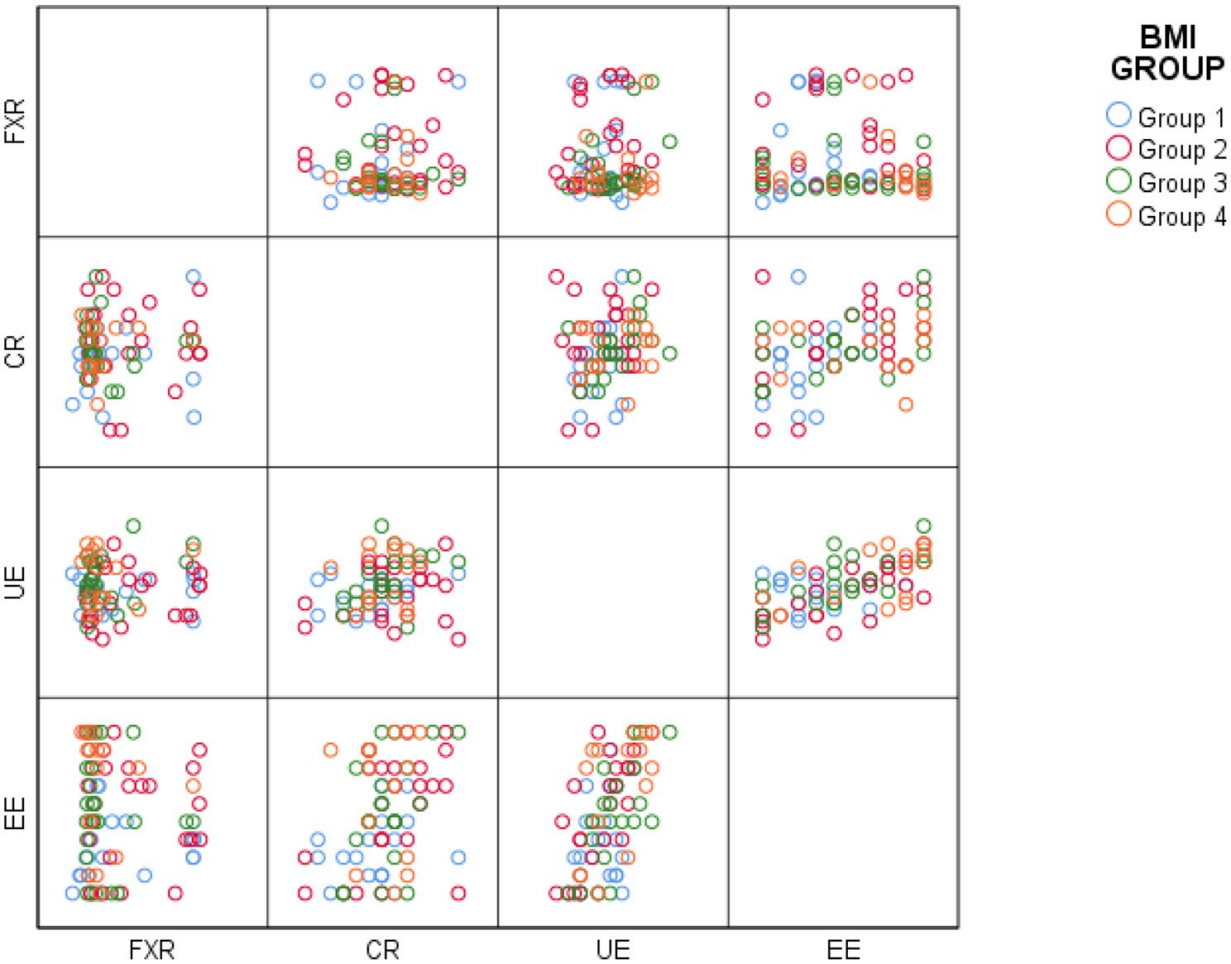
Pairwise scatter plots illustrating the relationships between serum FXR levels and TFEQ-R18 subscale scores. The figure presents pairwise scatter plots showing the relationships between serum Farnesoid X receptor (FXR) levels and the Three-Factor Eating Questionnaire–Revised 18 (TFEQ-R18) subscale scores, including cognitive restraint score (CRS), uncontrolled eating score (UES), and emotional eating score (EES). Each point represents an individual participant, and colors indicate BMI-based study groups: normal weight (Group 1), overweight (Group 2), obesity class I (Group 3), and obesity class II (Group 4)

Further analyses showed that serum FXR levels were not significantly associated with sex, comorbidities, physical activity status, regular sleep patterns, or eating behavior scores assessed by the TFEQ-R18, either in the overall cohort or within individual BMI groups. Nevertheless, the relationships between serum FXR levels and the TFEQ-R18 subscale scores are illustrated using a scatter plot–based visualization (Fig. 3).

In contrast, FXR levels were positively associated with a family history of obesity (*p* = 0.031) and the use of topical agents (*p* = 0.024). Among laboratory parameters, only serum zinc levels demonstrated a weak but statistically significant positive correlation with FXR concentrations (*r* = 0.292, *p* = 0.025).

## Discussion

The identification of FXR as a critical metabolic regulator has generated considerable interest in its potential as a therapeutic target for metabolic disorders, such as obesity [17]. In the present study, conducted in a cohort of healthy participants without chronic diseases or prior interventions, serum FXR levels differed significantly across BMI-defined categories. Specifically, individuals in the normal-weight group exhibited lower FXR concentrations compared with participants in higher BMI categories, suggesting that alterations in FXR levels are more apparent when adiposity is considered categorically rather than as a continuous measure. Consistent with this interpretation, no significant linear association was observed between BMI as a continuous variable and serum FXR levels, indicating that FXR regulation may reflect group-specific metabolic differences associated with increasing adiposity rather than a gradual, linear relationship with body weight. This distinction between categorical and continuous BMI analyses highlights the need to consider potential adiposity-related thresholds when interpreting FXR regulation.

Hepatic FXR expression is regulated by glucose and insulin metabolism [29] and is also present in pancreatic β-cells, where it suppresses postprandial glucose levels by inhibiting glycolysis [30]. In FXR-deficient mice, Ma et al. reported increased glycolytic and lipogenic gene expression, reduced gluconeogenesis, and the development of glucose intolerance and insulin resistance [31]. In our study, higher BMI was associated with progressively elevated blood glucose and HOMA-IR levels. These findings implicate reduced serum FXR levels in impaired glucose regulation and insulin resistance among individuals with higher BMI.

The relationship between zinc and insulin is complex, affecting both glucose homeostasis and insulin resistance. Although zinc supplementation has been proposed to improve glycemic control, evidence remains inconsistent and context-dependent [32]. In this study, serum FXR levels correlated weakly but significantly with blood zinc levels, suggesting that higher zinc may be associated with higher FXR. The mechanisms linking zinc, FXR, and insulin resistance remain unclear and highlight the need for further research.

The FXR-deficient mice model exhibits dyslipidemia, characterized by elevated serum glucose, impaired glucose and insulin tolerance, and increased LDL, TG, and HDL levels [21]. Consistent with these findings, our study demonstrated that individuals classified as first- and second-degree obese, who exhibited lower mean FXR levels, also presented with generally elevated mean levels of FBG, HOMA-IR, LDL, TG, and WBC levels compared to those with normal or lower body weight. These results suggest an association between serum FXR levels and obesity-related metabolic parameters, but they do not allow for firm conclusions regarding FXR expression or causality in metabolic dysregulation.

In the present study, no statistically significant sex-related differences were observed in serum FXR levels or associated metabolic parameters. This may be partly attributable to the limited sample size and cross-sectional design, which may have reduced the ability to detect subtle sex-specific effects. In addition, FXR regulation is influenced by metabolic status, bile acid signaling, and tissue-specific expression [33, 34], which may outweigh sex-related influences in clinically healthy populations. Accordingly, these findings should be interpreted with caution and do not exclude sex-specific FXR regulation under different physiological or pathological conditions.

Secondary bile acids synthesized by gut bacteria maintain intestinal barrier function through FXR signaling, underscoring the pivotal role of FXR in shaping the gut microbiota and maintaining intestinal homeostasis [35]. The relationship between serum FXR levels and BMI observed in our study contrasts with the association between intestinal FXR expression and BMI reported in previous literature. Previous studies have shown that obese individuals exhibit elevated intestinal FXR expression and heightened FXR signaling compared to lean controls [36, 37]. Although a causal relationship remains unclear, current evidence suggests tissue-specific regulation of FXR in obesity, characterized by reduced serum FXR and increased intestinal FXR activity. This highlights the complex, organ-specific roles of FXR in metabolic regulation across the liver, intestine, adipose tissue, and kidney. Hence, therapeutic strategies should focus on tissue-selective FXR modulation. Additionally, alterations in gut microbiota are well-documented in obesity, and studies have shown that microbiota-targeted interventions can suppress intestinal FXR signaling, thereby improving metabolic parameters [14, 17, 38]. In our study, serum FXR levels positively correlated with both a family history of obesity and the use of topical agents, partially supporting the influence of genetic and pharmacological factors on FXR regulation. Nonetheless, further research is needed to clarify the impact of microbiota-directed therapies on serum FXR in metabolic disorders.

As the participants’ BMI levels increased, the EE score averages from TFEQ-18 increased significantly and the degree of relationship was weak. This finding is similar to that of Keskitalo et al., who reported a stronger correlation between EES and BMI in overweight individuals compared to those with normal weight [39]. In addition, when the differences were examined in detail, it was found that the EE average of the normal weight group with high FXR was significantly lower than the average of the second degree obese group with high FXR. This observation suggests that emotional eating tendencies may increase with rising BMI, particularly in individuals with elevated FXR levels. Future studies should further investigate whether FXR-targeted interventions might also influence appetite regulation and emotional eating, particularly in populations with obesity.

This study has several limitations. Its cross-sectional design prevents causal inference between serum FXR levels and BMI, making it difficult to determine whether FXR activation constitutes a cause or a consequence of obesity. Although sample size was determined by power analysis, subgroup numbers may have limited the detection of subtle associations. Additionally, reliance on self-reported data for lifestyle and eating behaviors may introduce bias. Furthermore, the absence of direct bile acid measurements and comprehensive dietary assessments may have limited a more detailed interpretation of the FXR–obesity relationship. Future research employing longitudinal designs and incorporating tissue-specific and microbiome analyses may help to further elucidate FXR-related metabolic pathways.

## Conclusion

In this prospective cross-sectional study, serum FXR levels were examined in normal-weight, overweight, and obese individuals, with statistically significant differences observed among the groups. These findings suggest that serum FXR may serve as a potential regulatory target for metabolic disorders associated with obesity. However, to confirm FXR as a definitive biomarker and therapeutic target in metabolic disease management, large-scale longitudinal studies and clinical trials are warranted. Additionally, if serum FXR is to be considered for therapeutic use, its long-term safety and efficacy must be thoroughly evaluated to ensure clinical applicability.

## Supplementary Information

Below is the link to the electronic supplementary material.

Supplementary Material 1

## Data Availability

The datasets generated and/or analysed during the current study are not publicly available due to participant confidentiality but are available from the corresponding author on reasonable request.

## Abbreviations

FXR: Farnesoid X Receptor
BMI: Body Mass Index
TFEQ: Three-Factor Eating Questionnaire
CR: Cognitive Restraint
UE: Uncontrolled Eating
EE: Emotional Eating
FBG: Fasting Blood Glucose
HOMA-IR: Homeostatic Model Assessment of Insulin Resistance
LDL: Low-Density Lipoprotein
TG: Triglyceride
AST: Aspartate Aminotransferase
ALT: Alanine Aminotransferase
WBC: White Blood Cell
NLR: Neutrophil-to-Lymphocyte Ratio
RBC: Red Blood Cell
HGB: Haemoglobin
HCT: Haematocrit
MCV: Mean Corpuscular Volume
PLT: Platelet
MPV: Mean Platelet Volume
Zn: Serum Zinc
Fe: Serum Iron
Mg: Serum Magnesium
TSH: Thyroid-Stimulating Hormone

## References

[1] Safaei M, Sundararajan EA, Driss M, Boulila W, Shapi’i A. A systematic literature review on obesity: Understanding the causes & consequences of obesity and reviewing various machine learning approaches used to predict obesity. Comput Biol Med. 2021;136:104754. 10.1016/j.compbiomed.2021.104754. 34426171 10.1016/j.compbiomed.2021.104754

[2] Kelly T, Yang W, Chen CS, Reynolds K, He J. Global burden of obesity in 2005 and projections to 2030. Int J Obes (Lond). 2008;32(9):1431–7. 10.1038/ijo.2008.102. 18607383 10.1038/ijo.2008.102

[3] De Magalhaes Filho CD, Downes M, Evans RM. Farnesoid X receptor: an emerging target to combat obesity. Dig Dis. 2017;35(3):185–90. 10.1159/000450909. 28249279 10.1159/000450909 PMC5417073

[4] Zhang Y, Kast-Woelbern HR, Edwards PA. Natural structural variants of the nuclear receptor farnesoid X receptor affect transcriptional activation. J Biol Chem. 2003;278(1):104–10. 10.1074/jbc.M209505200. 12393883 10.1074/jbc.M209505200

[5] Kuipers F, Bloks VW, Groen AK. Beyond intestinal soap—bile acids in metabolic control. Nat Rev Endocrinol. 2014;10(8):488–98. 10.1038/nrendo.2014.60. 24821328 10.1038/nrendo.2014.60

[6] Halilbasic E, Claudel T, Trauner M. Bile acid transporters and regulatory nuclear receptors in the liver and beyond. J Hepatol. 2013;58(1):155–68. 10.1016/j.jhep.2012.08.002. 22885388 10.1016/j.jhep.2012.08.002 PMC3526785

[7] Chávez-Talavera O, Tailleux A, Lefebvre P, Staels B. Bile acid control of metabolism and inflammation in obesity, type 2 diabetes, dyslipidemia, and nonalcoholic fatty liver disease. Gastroenterology. 2017;152(7):1679–94. 10.1053/j.gastro.2017.01.055. 28214524 10.1053/j.gastro.2017.01.055

[8] Cariou B, van Harmelen K, Duran-Sandoval D, van Dijk TH, Grefhorst A, Abdelkarim M, Caron S, Torpier G, Fruchart JC, Gonzalez FJ, et al. The farnesoid X receptor modulates adiposity and peripheral insulin sensitivity in mice. J Biol Chem. 2006;281(17):11039–49. 10.1074/jbc.M510258200. 16446356 10.1074/jbc.M510258200

[9] Prawitt J, Abdelkarim M, Stroeve JHM, Popescu I, Duez H, Velagapudi VR, Dumont J, Bouchaert E, van Dijk TH, Lucas A, et al. Farnesoid X receptor deficiency improves glucose homeostasis in mouse models of obesity. Diabetes. 2011;60(7):1861–71. 10.2337/db11-0030. 21593203 10.2337/db11-0030 PMC3121443

[10] Zhang Y, Castellani LW, Sinal CJ, Gonzalez FJ, Edwards PA. Loss of FXR protects against diet-induced obesity and accelerates liver carcinogenesis in ob/ob mice. Mol Endocrinol. 2012;26(2):272–80. 10.1210/me.2011-1157. 22261820 10.1210/me.2011-1157 PMC3275160

[11] Mueller M, Thorell A, Claudel T, Jha P, Koefeler H, Lackner C, Hoesel B, Fauler G, Stojakovic T, Einarsson C, et al. Ursodeoxycholic acid exerts farnesoid X receptor-antagonistic effects on bile acid and lipid metabolism in morbid obesity. J Hepatol. 2015;62(6):1398–404. 10.1016/j.jhep.2014.12.034. 25617503 10.1016/j.jhep.2014.12.034 PMC4451470

[12] Chiang JY, Ferrell JM. Bile acid metabolism in liver pathobiology. Gene expr. 2018;18(2):71. 10.3727/105221618X15156018385515. 29325602 10.3727/105221618X15156018385515 PMC5954621

[13] Ding L, Yang Q, Zhang E, Wang Y, Sun S, Yang Y, Tian T, Ju Z, Jiang L, Wang X, et al. Notoginsenoside Ft1 acts as a TGR5 agonist but FXR antagonist to alleviate high fat diet-induced obesity and insulin resistance in mice. APSB. 2021;11(6):1541–54. 10.1016/j.apsb.2021.03.038. 10.1016/j.apsb.2021.03.038 PMC8245856 34221867

[14] Li Y, Wang L, Yi Q, Luo L, Xiong Y. Regulation of bile acids and their receptor FXR in metabolic diseases. Front Nutr. 2024;11:1447878. 10.3389/fnut.2024.1447878. 39726876 10.3389/fnut.2024.1447878 PMC11669848

[15] Panzitt K, Jungwirth E, Vosko LE, Madreiter-Sokolowski CT, Madl T, Tawfik I, Habisch H, Krstic J, Prokesch A, Karitnig R, et al. FXR adapts hepatic mitochondrial function to increased substrate oxidation in patients with obesity. Sci Trans Med. 2025;17(811):eadn4558. 10.1126/scitranslmed.adn4558. 10.1126/scitranslmed.adn4558 40802742

[16] Sun L, Cai J, Gonzalez FJ. The role of farnesoid X receptor in metabolic diseases, and gastrointestinal and liver cancer. Nat Rev Gastroenterol Hepatol. 2021;18(5):335–47. 10.1038/s41575-020-00404-2. 33568795 10.1038/s41575-020-00404-2

[17] Chiang JY, Ferrell JM. Discovery of farnesoid X receptor and its role in bile acid metabolism. Mol Cell Endocrinol. 2022;548:111618. 10.1016/j.mce.2022.111618. 35283218 10.1016/j.mce.2022.111618 PMC9038687

[18] Jiang C, Xie C, Lv Y, Li J, Krausz KW, Shi J, Brocker CN, Desai D, Amin SG, Bisson WH, et al. Intestine-selective farnesoid X receptor inhibition improves obesity-related metabolic dysfunction. Nat Commun. 2015;6:10166. 10.1038/ncomms10166. 26670557 10.1038/ncomms10166 PMC4682112

[19] Tang Q, Wang C, Jin G, Hou H, Wang X, Guo Q, Liu T, Wang S, Dai X, Wang B. Early life dietary emulsifier exposure predisposes the offspring to obesity through gut microbiota-FXR axis. Int Food Res J. 2022;162:111921. 10.1016/j.foodres.2022.111921. 10.1016/j.foodres.2022.111921 36461273

[20] Ryan KK, Tremaroli V, Clemmensen C, Kovatcheva-Datchary P, Myronovych A, Karns R, Wison- Pérez HE, Sandoval DA, Kohli R, Bäckhed F, et al. FXR is a molecular target for the effects of vertical sleeve gastrectomy. Nature. 2014;509(7499):183–8. 10.1038/nature13135. 24670636 10.1038/nature13135 PMC4016120

[21] Chiang JY, Ferrell JM. Bile acid receptors FXR and TGR5 signaling in fatty liver diseases and therapy. Am J Physiol Gastrointest Liver Physiol. 2020;318(3):G554–73. 10.1152/ajpgi.00223.2019. 31984784 10.1152/ajpgi.00223.2019 PMC7099488

[22] Bull FC, Al-Ansari SS, Biddle S, Borodulin K, Buman MP, Cardon G, Carty C, Chaput JP, Chastin S, Chou R, et al. World Health Organization 2020 guidelines on physical activity and sedentary behaviour. Br J Sports Med. 2020;54(24):1451–62. 10.1136/bjsports-2020-102955. 33239350 10.1136/bjsports-2020-102955 PMC7719906

[23] Watson NF, Badr MS, Belenky G, Bliwise DL, Buxton OM, Buysse D, Dinges DF, Gangwisch J, Grandner MA, Kushida C, et al. Recommended amount of sleep for a healthy adult: A joint consensus statement. Sleep. 2015;38(6):843–4. 10.5664/jcsm.4758. 26039963 10.5665/sleep.4716 PMC4434546

[24] Stunkard AJ, Messick S. The three-factor eating questionnaire to measure dietary restraint, disinhibition and hunger. J Psychosom Res. 1985;29:71–83. 10.1016/0022-3999(85)90010-8. 3981480 10.1016/0022-3999(85)90010-8

[25] Hyland ME, Irvine SH, Thacker C, Dann PL, Dennis I. Psychometric analysis of the Stunkard-Messick Eating Questionnaire (SMEQ) and comparison with the Dutch Eating Behavior Questionnaire (DEBQ). Curr Psychol Res Rev. 1989;8:228–33. 10.1007/BF02686751.

[26] Karlsson J, Persson LO, Sjostrom L, Sullivan M. Psychometric properties and factor structure of the Three-Factor Eating Questionnaire (TFEQ) in obese men and women. Results from the Swedish Obese Subjects (SOS) study. Int J Obes Relat Metab Disord. 2000;24:1715–25. 10.1038/sj.ijo.0801442. 11126230 10.1038/sj.ijo.0801442

[27] Lauzon B, Romon M, Deschamps V, Lafay L, Borys JM, Karlsson J, Ducimetie`re P, Charles MA, Fleurbaix Laventie Ville Sante (FLVS) Study Group. The Three-Factor Eating Questionnaire-R18 Is Able to Distinguish amongDifferent Eating Patterns in a General Population. J Nutr. 2004;134:2372–80. 10.1093/jn/134.9.2372. 15333731 10.1093/jn/134.9.2372

[28] Kıraç D, Kaspar EÇ, Avcılar T, Çakır ÖK, Ulucan K, Kurtel H, Deyneli O, Güney Aİ. A new method in investigation of obesity-related eating behaviors ‘three-factor eating questionnaire’. Clin Exp Health Sci. 2015;5(3):162–9. 10.5455/musbed.20150602015512.

[29] Duran-Sandoval D, Cariou B, Percevault F, Hennuyer N, Grefhorst A, van Dijk TH, Gonzalez FJ, Fruchart JC, Kuipers F, Staels B. The farnesoid X receptor modulates hepatic carbohydrate metabolism during the fasting-refeeding transition. J Biol Chem. 2005;280(33):29971–9. 10.1074/jbc.M501931200. 15899888 10.1074/jbc.M501931200

[30] Renga B, Mencarelli A, Vavassori P, Brancaleone V, Fiorucci S. The bile acid sensor FXR regulates insulin transcription and secretion. Biochim Biophys Acta. 2010;1802(3):363–72. 10.1016/j.bbadis.2010.01.002. 20060466 10.1016/j.bbadis.2010.01.002

[31] Ma K, Saha PK, Chan L, Moore DD. Farnesoid X receptor is essential for normal glucose homeostasis. J Clin Invest. 2006;116(4):1102–9. 10.1172/JCI25604. 16557297 10.1172/JCI25604 PMC1409738

[32] Kim J, Lee S. Effect of zinc supplementation on insulin resistance and metabolic risk factors in obese Korean women. Nutr Res Pract. 2012;6(3):221–5. 10.4162/nrp.2012.6.3.221. 22808346 10.4162/nrp.2012.6.3.221 PMC3395787

[33] Lefebvre P, Cariou B, Lien F, Kuipers F, Staels B. Role of bile acids and bile acid receptors in metabolic regulation. Physiol Rev. 2009;89(1):147–91. 10.1152/physrev.00010.2008. 19126757 10.1152/physrev.00010.2008

[34] Chiang JYL. Bile acid metabolism and signaling. Compr Physiol. 2013;3(3):1191–212. 10.1002/cphy.c120023. 23897684 10.1002/cphy.c120023 PMC4422175

[35] Kakiyama G, Marques D, Takei H, Nittono H, Erickson S, Fuchs M, Rodriguez-Agudo D, Gil G, Hylemon PH, Zhou H, et al. Mitochondrial oxysterol biosynthetic pathway gives evidence for CYP7B1 as controller of regulatory oxysterols. J Steroid Biochem Mol Biol. 2019;189:36–47. 10.1016/j.jsbmb.2019.01.011. 30710743 10.1016/j.jsbmb.2019.01.011

[36] Friedman SL. Mechanisms of hepatic fibrogenesis. Gastroenterology. 2008;134(6):1655–69. 10.1053/j.gastro.2008.03.003. 18471545 10.1053/j.gastro.2008.03.003 PMC2888539

[37] Zhang L, Xie C, Nichols RG, Chan SH, Jiang C, Hao R, Smith PB, Cai J, Simons MN, Hatzakis E, et al. Farnesoid X receptor signaling shapes the gut microbiota and controls hepatic lipid metabolism. MSystems. 2016;1(5):10–1128. 10.1128/msystems.00070-16. 10.1128/mSystems.00070-16 PMC5080402 27822554

[38] Jiang J, Fan M, Yuan W, Yue D, Wang Z, Yang L, Huang W, Jin L, Wang X, Ding L. Hepatic and intestinal tissue-specific Fxr deficiency alters bile acid homeostasis in female mice. Am J Physiology-Gastrointestinal Liver Physiol. 2025;328(6):G774–90. 10.1152/ajpgi.00387.2024. 10.1152/ajpgi.00387.2024 40338063

[39] Keskitalo K, Tuorila H, Spector TD, Cherkas LF, Knaapila A, Kaprio J, Silventoinen K, Perola M. The Three-Factor Eating Questionnaire, body mass index, and responses to sweet and salty fatty foods: a twin study of genetic and environmental associations. AJCN. 2008;88(2):263–71. 10.1093/ajcn/88.2.263. 10.1093/ajcn/88.2.263 18689360

